# Preliminary Changes in Head Control and Segmental Trunk Control Following an Eight-Week Core Strengthening Program in Children with Bilateral Spastic Cerebral Palsy

**DOI:** 10.3390/healthcare14142170

**Published:** 2026-07-18

**Authors:** Afnan M. Alkhateeb, Shehab M. Abd El-Kader, Alanoud K. Alsulami, Tamer M. El-Saeed

**Affiliations:** 1 Department of Physical Therapy, Faculty of Medical Rehabilitation Sciences, King Abdulaziz University, Jeddah 22252, Saudi Arabia; 2 Department of Physical Therapy for Cardiopulmonary Disorders and Geriatrics, Faculty of Physical Therapy, Cairo University, Giza 12613, Egypt; 3 Department of Physical Therapy for Pediatrics, Faculty of Physical Therapy, Cairo University, Giza 12613, Egypt

**Keywords:** cerebral palsy, trunk control, head control, core strengthening, segmental assessment of trunk control

## Abstract

**Background:** Children with bilateral spastic cerebral palsy classified at the more severe levels of the Gross Motor Function Classification System (GMFCS) exhibit profound deficits in head and trunk control that constrain functional independence, feeding safety, and caregiver burden. **Objective:** To characterize within-subject changes in head control and segmental trunk control following an eight-week, manualized, therapist-supervised core muscle strengthening program in children with bilateral spastic cerebral palsy at GMFCS levels IV and V. **Methods:** A single-arm quasi-experimental study with within-subject assessments at baseline, mid-intervention, and post-intervention (week 8) was conducted. Following removal of two participants at the data-cleaning stage for a baseline eligibility deviation, 36 children aged 4–10 years received a structured core strengthening program delivered three times weekly for eight consecutive weeks (24 sessions total, 45–50 min each). The two standardized outcome measures were the Head Control Scale (HCS) and the Segmental Assessment of Trunk Control (SATCo), the latter analyzed as Static subscale, Active subscale, and Total composite. **Results:** Statistically significant improvement over time was observed on every outcome (all Friedman *p* < 0.001; Kendall’s W range 0.813–0.950), and every pairwise interval comparison was significant after Bonferroni adjustment. Pre-to-post change reached large to very large effect sizes. As a secondary robustness check, one-way repeated-measures analysis of variance corroborated effects of time on the HCS and the SATCo Static and Total composites (all *p* < 0.001; partial η^2^ range 0.722–0.792). **Conclusions:** These preliminary findings suggest that the program is feasible and associated with statistically significant within-subject improvements in head and trunk control in children with bilateral spastic cerebral palsy at GMFCS levels IV and V. Because the study had no control group, outcomes were confined to the body-functions level, and maintenance was not assessed, causal inference and translation to activity and participation remain undetermined; controlled trials with follow-up are required to determine efficacy.

## 1. Introduction

Cerebral palsy (CP) is the most common physical disability of childhood, defined as a group of permanent disorders of movement and posture arising from non-progressive disturbances of the developing fetal or infant brain [1,2]. The accompanying impairments commonly extend beyond the motor domain to include disturbances of sensation, perception, cognition, communication, and behavior, alongside seizures and secondary musculoskeletal complications [3,4]. The global prevalence of cerebral palsy is estimated at approximately 1.5–3.0 affected children per 1000 live births [5], with broadly comparable figures emerging from Arab-region investigations, including a Jeddah-based study reporting 2.1 per 1000 [6]. Bilateral spastic presentations represent the most disabling subgroup and disproportionately occupy the more severe levels of the Gross Motor Function Classification System (GMFCS) [7].

Children classified at GMFCS levels IV and V are by definition incapable of independent walking. Their everyday functioning is heavily dependent on caregivers for mobility, transfers, hygiene, feeding, and positioning. Within this population, head and trunk control deficits are central determinants of functional and quality-of-life outcomes: stable head control supports safe oral intake and swallowing [8] and underpins eye-gaze-based communication in children with severe cerebral palsy [9], while trunk stability provides the proximal foundation for any transitional movement, supported sitting, or distal upper-limb function [10]. Families of non-ambulant children consistently identify postural comfort, ease of daily care, and functional positioning gains among their highest treatment priorities [11].

From a developmental-neuroscience standpoint, postural control is acquired in a cephalocaudal hierarchy. Stable head control emerges first, followed by progressive recruitment of segmental trunk musculature from the upper thoracic level downward through the lumbar spine until sitting balance and ultimately upright standing become possible [12]. The trunk in this framework is not a passive substrate but an actively organized control system that anticipates limb movements through feed-forward postural adjustments, accommodates gravitational demands of changing positions, and responds reactively to perturbations [13,14]. In children with bilateral spastic cerebral palsy, the sequence is disrupted by a complex confluence of spasticity, muscle weakness, altered sensory processing, impaired motor planning, and secondary musculoskeletal constraints [15,16]. Without stable proximal control, head stability suffers, sitting cannot be sustained without external support, and active distal function becomes inefficient or impossible [17].

Theoretical and clinical traditions have long proposed that targeted strengthening and activation of core musculature should yield benefits that propagate proximally to distally, improving head control as a downstream consequence of improved trunk stability [18]. A randomized trial of segmental trunk support during therapy in children with moderate-to-severe cerebral palsy demonstrated greater gains in trunk and reaching control than conventional therapy alone [19]. A core strengthening program in children with hemiplegic cerebral palsy yielded significant improvements in trunk endurance and gait parameters [20]. A 2025 meta-analysis of trunk-targeted interventions across the cerebral palsy spectrum reported gains in trunk control and gross motor function but pooled heterogeneous studies of variable quality and explicitly noted the under-representation of severely affected non-ambulant children; its conclusions should therefore be read with that heterogeneity in mind [21].

The severely affected non-ambulant phenotype—precisely the group for whom head and trunk control deficits are most disabling—is the subgroup for whom intervention evidence is sparsest, and the magnitude and trajectory of response to a structured trunk-targeted program have not been well characterized within this group.

The present investigation was designed to address these gaps. The primary objective was to characterize within-subject changes in head control and segmental trunk control in children aged 4–10 years with bilateral spastic cerebral palsy at GMFCS levels IV and V following an eight-week, manualized core muscle strengthening program. A single-arm quasi-experimental design with pre-, mid-, and post-intervention assessments was chosen because the severely affected non-ambulant population is a clinical priority group for whom withholding active rehabilitation is ethically problematic, and because no prior trial has characterized the magnitude or trajectory of response in this specific phenotype; the design supports inferences about within-subject change rather than causal attribution and is intended to generate effect-size estimates to power a subsequent randomized controlled trial.

## 2. Materials and Methods

### 2.1. Study Design

This study was conducted as a prospective, single-arm, quasi-experimental investigation with within-subject pre-intervention (week 0), mid-intervention (week 4), and post-intervention (week 8) assessments. The design was selected to provide a within-subject pre–post comparison of change over the intervention period while remaining ethically and operationally appropriate for a severely affected non-ambulant pediatric population for whom withholding active rehabilitation would be problematic. An active comparator (for example, dose-matched conventional physiotherapy), a usual-care comparator, or a delayed-start design would have permitted stronger inference; these were not adopted owing to the limited pool of eligible GMFCS IV–V children at the two participating centres and the resource and timeline constraints of a first characterization study, so the design supports inferences about within-subject change rather than causal attribution. The target sample was pragmatic, comprising all consecutive eligible children over the recruitment window, and the study was intended to estimate within-subject effect sizes to inform a subsequent randomized controlled trial rather than to test a hypothesis at fixed statistical power. Reporting follows the Transparent Reporting of Evaluations with Nonrandomized Designs (TREND) statement [22] and incorporates Template for Intervention Description and Replication (TIDieR) elements [23].

### 2.2. Setting

Recruitment and intervention delivery took place at the outpatient pediatric rehabilitation departments of King Abdulaziz Hospital (Al-Mahjar) and Al-Aziziyah Children’s Hospital in Jeddah, Saudi Arabia, both tertiary referral centers for pediatric neuromotor disability. Recruitment commenced on 1 May 2025, and each child was assessed at baseline (week 0) and at 4 and 8 weeks relative to their individual start date. Fifteen children were recruited at King Abdulaziz Hospital (Al-Mahjar) and 21 at Al-Aziziyah Children’s Hospital. Registered on 29 May 2026 (recruitment status: Completed), registration was retrospective. This trial was registered retrospectively at ClinicalTrials.gov (NCT07613515; registered May 2026); participant enrolment commenced on May 2025. The study derives from an MSc thesis, and the candidate—registering a trial for the first time—was not fully aware of the registry submission process, so an active registration was not completed until the manuscript was prepared. No changes were made to the eligibility criteria, intervention, or pre-specified primary outcomes (the Head Control Scale and the Segmental Assessment of Trunk Control) between enrolment and registration, and the retrospective registration did not affect the design, conduct, analysis, or reporting of the study. This manuscript reports the registered primary outcomes (HCS and SATCo); the Gross Motor Function Measure-66 (GMFM-66) and the Caregiver Priorities and Child Health Index of Life with Disabilities (CPCHILD) were designated for future work and are not reported here. The present report is deliberately limited to characterizing body-function-level change in the registered primary outcomes; the activity- and participation-level measures (GMFM-66 and CPCHILD) and clinical righting reactions require dedicated analysis and are reserved for a separate study.

### 2.3. Ethical Approval and Informed Consent

The study protocol was reviewed and approved by the Research Ethics Committee of the Faculty of Medicine, King Abdulaziz University (National Committee of Bioethics Registration Number: HA-02-J-008) with ethical approval reference number: 304-25, and conducted in accordance with the principles of the Declaration of Helsinki. Written informed consent was obtained from the parent or legal guardian of every participating child prior to any study-related procedure. The voluntary nature of participation, the right to withdraw at any time without prejudice to ongoing care, and the data-handling and confidentiality provisions were explained verbally and in writing by a member of the research team.

### 2.4. Participants

Children were eligible for inclusion if they met all of the following criteria: a documented diagnosis of bilateral spastic cerebral palsy made by a pediatric neurologist or physiatrist; age between 4 and 10 years inclusive; classification at GMFCS level IV or V on independent assessment by two trained pediatric physiotherapists, with discrepant ratings resolved by consensus; incomplete head control in supported sitting at baseline, defined as inability to maintain the head in midline against gravity for 30 s when supported at the upper thoracic level; and parent or legal guardian able and willing to provide written informed consent and to commit to thrice-weekly attendance over the eight-week intervention period.

Children were excluded if they met any of the following criteria: orthopedic surgery involving the trunk, hip, or pelvic region within the preceding six months; botulinum toxin injections to the trunk or proximal lower limb musculature within three months prior to enrolment; active uncontrolled seizures defined as more than one seizure per week despite optimized antiepileptic therapy, or other severe medical instability precluding intensive therapy participation; fixed structural scoliosis with a Cobb angle exceeding 40 degrees; visual or auditory impairments severe enough to preclude reliable engagement with assessment procedures; or concurrent enrolment in another interventional study.

### 2.5. Intervention

#### 2.5.1. Overall Structure and Dose

All participants received the same eight-week intervention delivered as three 45–50 min sessions per week, yielding a planned dose of 24 sessions per participant over the eight-week period. Each session was structured into a 5 min preparatory phase, a 35–40 min structured core block, and a 5 min cool-down phase. Sessions were delivered on a one-to-one basis by pediatric physiotherapists with at least five years of clinical experience in cerebral palsy, all of whom had completed an eight-hour training workshop on the trial intervention protocol prior to commencing recruitment.

#### 2.5.2. Preparatory Phase

The 5 min preparatory phase incorporated gentle passive range-of-motion exercises for the major joints of the lower limb and trunk, prolonged stretching of hip flexors, hamstrings, and adductors held for approximately 30 s per muscle group, and tactile-proprioceptive input to the trunk delivered through firm but gentle pressure along the paraspinal musculature. The purpose of this phase was to prepare the musculoskeletal system for the core block by reducing reflex hyperexcitability, optimizing tissue extensibility, and providing afferent input to facilitate subsequent active engagement.

#### 2.5.3. Core Strengthening Block

The 35–40 min core block consisted of four manualized activities delivered in a structured rotation:

1. Therapist-assisted bridging in supine. Beginning with manual facilitation of pelvic elevation off the supporting surface and progressing to less-assisted holds of 5–15 s duration, with the aim of activating the gluteal and lumbar paraspinal musculature in a closed-chain pattern [24].
2. Modified quadruped position over a graduated bolster. Therapist support was provided at the pelvis with verbal and tactile cueing to encourage active trunk extension and weight-bearing through the upper limbs. Bolster height was decreased progressively across the eight weeks to increase the demand on trunk extensor activation against gravity [25].
3. Supported sitting on a 45–55 cm therapy ball with multidirectional perturbations. The therapist provided pelvic stabilization and applied graded perturbations to elicit reactive trunk and head responses, beginning with slow predictable anterior–posterior displacements and progressing across weeks to faster medio-lateral and combined perturbations [26].
4. Assisted pull-to-sit from supine. Upper-limb support was progressively reduced over the eight-week period with the aim of eliciting progressively greater anterior trunk and head flexor activation against gravity [27].

Progressions in repetitions, hold durations, and perturbation difficulty were specified in a written intervention manual and were signed off weekly by the supervising physiotherapist for each participant. The four-activity rotation was preserved across the eight-week intervention; what varied was the difficulty level within each activity according to written progression criteria.

#### 2.5.4. Cool-Down Phase

The closing 5 min cool-down comprised passive cool-down stretching of the muscle groups loaded during the core block and supported positioning to allow the child to return to baseline arousal level prior to leaving the session.

#### 2.5.5. Treatment Fidelity and Adherence Monitoring

Treatment fidelity was monitored by an independent physiotherapist who reviewed randomly selected 20% of all video-recorded sessions (164 of 822 delivered sessions) using a 12-item protocol-adherence checklist, each item scored dichotomously as delivered or not delivered as specified. Fidelity was expressed as the proportion of items delivered, both per session and across all reviewed sessions, and adequate fidelity was defined as a priori as delivery of at least 80% of items per session. Sessions were selected using a random-number generator and distributed across therapists and across the three study phases.

Adherence was operationalized as the number of the 24 prescribed sessions attended by each child, expressed as a percentage and summarized as mean ± standard deviation and range; the pre-specified per-protocol threshold was attendance at ≥75% of sessions (≥18 of 24). All participants received the same usual care (a standard home program) during the study period, and no concomitant botulinum toxin, orthotic, or medication changes occurred during the eight-week intervention.

### 2.6. Outcome Measures

Two standardized outcome measures were administered and reported at baseline, mid-intervention (week 4), and post-intervention (week 8) by physiotherapists trained in the relevant standardized assessment procedures. All assessments were conducted at the same time of day and in the same clinical environment for each participant to minimize circadian and contextual sources of measurement variability.

Assessments were scored live rather than from masked video recordings, and assessors were not blinded to timepoint or intervention receipt; this is a potential source of detection bias and is revisited in the Limitations. No formal inter-rater calibration, duplicate scoring, or intra-rater reliability assessment was undertaken within the present study, which is acknowledged as a measurement limitation.

#### 2.6.1. Head Control Scale (HCS)

The Head Control Scale used in this study comprised three position-based items—supine, prone, and supported sitting—each scored 0–2 according to written behavioral descriptors, with the three item scores summed to produce an HCS Total ranging from 0 (no head control in any position) to 6 (full age-appropriate head control across all three positions). Head control was operationalized as the ability to maintain the head in a stable midline position against gravity for at least 30 s during the positional test [28].

#### 2.6.2. Segmental Assessment of Trunk Control (SATCo)

The Segmental Assessment of Trunk Control [29] provides a hierarchical, segmental evaluation of trunk control by progressively reducing manual support from the shoulder girdle downward through seven anatomical levels: head, upper thoracic, mid-thoracic, lower thoracic, upper lumbar, lower lumbar, and full trunk. At each segmental level, two categories of control were tested in this study: static (steady-state holding) and active (anticipatory control during voluntary head turning). Each item was scored dichotomously (1 = control present, 0 = absent), yielding two subscale scores ranging from 0 to 7 (SATCo Static and SATCo Active) and a Total composite ranging from 0 to 14 (Static + Active). The SATCo has demonstrated good-to-excellent inter-rater and intra-rater reliability (intraclass correlation > 0.80) in children with neuromotor disability and responsiveness to change across rehabilitation interventions [30].

Clinical righting reactions were also recorded at all three timepoints with complete data but were not among the pre-specified primary outcomes of this report; they are reserved for a separate analysis and are not presented here. All other outcomes were assessed at all three timepoints with complete data for every enrolled participant.

### 2.7. Statistical Analysis

All analyses were performed with the final analyzed sample (N = 36). Because there was no loss to follow-up and no missing outcome data, no imputation was required. Continuous outcomes are reported as mean ± standard deviation and as median with interquartile range. Normality of the distribution of each outcome at each timepoint was evaluated using the Shapiro–Wilk test and visual inspection of quantile-quantile plots; given the ordinal nature of the composite scores, non-parametric tests were adopted as the primary inferential approach, with parametric repeated-measures analysis of variance reported only as a secondary robustness check and is interpreted with caution, and was not applied to the SATCo Active subscale, whose baseline floor (mean 0.00, SD 0.00) precludes meaningful parametric modelling. A sensitivity analysis including the two participants removed at data cleaning (N = 38) is provided in Supplementary File S1, in which the direction and statistical significance of all findings were unchanged.

For each outcome, the Friedman non-parametric repeated-measures test was applied across the three timepoints as the omnibus test for change over time, with the Kendall coefficient of concordance (W) reported as the effect-size index. Significant omnibus tests were followed by pairwise Wilcoxon signed-rank tests for the three timepoint contrasts (week 0 versus week 4; week 0 versus week 8; week 4 versus week 8), with the significance threshold tightened to α = 0.017 via Bonferroni adjustment to control the family-wise Type I error rate across three comparisons. Effect sizes for the pairwise comparisons were quantified using Cohen’s dz (the mean change divided by the standard deviation of the change scores) and the Wilcoxon r (the standardized test statistic divided by the square root of the sample size), with conventional benchmarks of 0.20 (small), 0.50 (medium), and 0.80 (large) for Cohen’s dz. As a parametric corroboration, one-way repeated-measures analysis of variance was conducted with time as the within-subjects factor, with sphericity evaluated using Mauchly’s test and partial eta-squared (η^2^p) reported as the effect-size index. A two-tailed α of 0.05 was used unless otherwise specified, and analyses were performed using IBM SPSS software v27.

## 3. Results

### 3.1. Participant Characteristics and Flow

**Figure 1:**
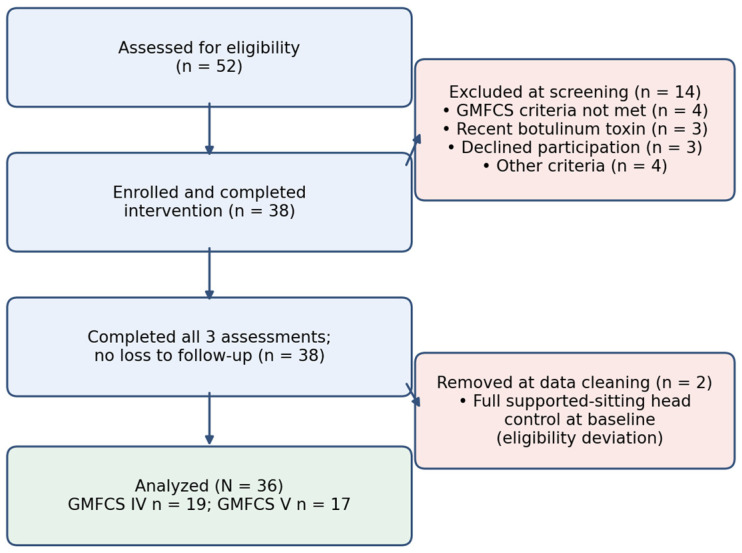
Participant flow through the study (52 screened; 14 excluded at screening; 38 enrolled; 2 removed at data cleaning for baseline eligibility deviation; 36 analyzed).

Of 52 children screened, 14 were excluded at screening (four did not meet GMFCS criteria, three had received recent botulinum toxin injections, three declined participation, and four did not meet other inclusion criteria), leaving 38 who were enrolled and completed the eight-week intervention and all three assessments with no loss to follow-up (Figure 1). At the data-cleaning stage, two participants were found to have had full head control on the supported-sitting item at baseline—contrary to the eligibility criterion of incomplete supported-sitting head control—and were removed, yielding a final analyzed sample of 36 children. After removal, all analyzed participants had an HCS baseline of exactly 3/6.

The analyzed sample comprised 20 boys and 16 girls aged 4–10 years (mean 6.94, SD 2.01). Nineteen children were classified at GMFCS level IV (52.8%) and 17 at GMFCS level V (47.2%); 27 children (75.0%) had spastic quadriplegic distribution and 9 (25.0%) had spastic diplegic distribution. Baseline characteristics, including scores by GMFCS level, are summarized in Table 1.

### 3.2. Adherence and Adverse Events

Adherence to the prescribed dose was high. Participants attended a mean of 22.8 ± 1.0 of the 24 planned sessions (median 23; range 20–24), corresponding to 95.1% overall adherence, and every participant exceeded the pre-specified 75% per-protocol threshold (minimum individual adherence 83.3%). Adverse events were monitored at every session and defined a priori as any untoward physical or behavioral event temporally associated with the intervention (for example, pain, spasticity exacerbation, excessive fatigue, or irritability), graded as mild (transient and self-resolving), moderate (requiring session modification), or serious (requiring medical attention or session termination). No serious adverse events occurred. Mild adverse events, transient post-session fatigue or irritability, all self-resolving were recorded in 7 of 36 children (11 events in total). All events were graded mild; no moderate events occurred, no session modifications were required, and no participant required premature termination. Treatment fidelity was high, with all reviewed sessions meeting the pre-specified 80% adequacy threshold for delivery of the manualized protocol items.

### 3.3. Head and Trunk Control Outcomes

**Figure 2:**
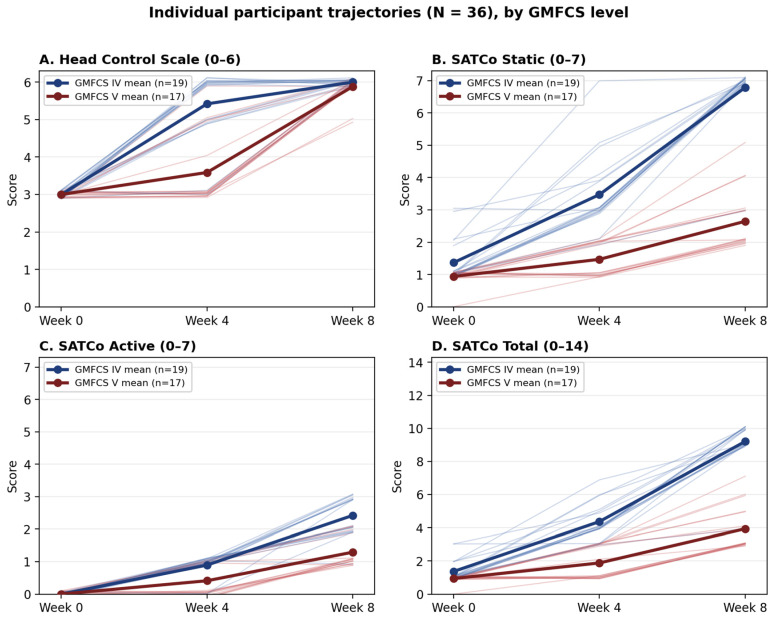
Individual participant trajectories (N = 36) for each outcome across the three assessment timepoints, with GMFCS-level means overlaid (blue = GMFCS IV; red = GMFCS V). Thin lines are individual children (jittered for visibility).

Descriptive statistics for the four outcomes at each assessment timepoint are presented in Table 2, and the mean trajectories are displayed in Figure 2. Every outcome improved monotonically from week 0 through week 4 to week 8. At baseline all 36 analyzed participants scored 3/6 on the HCS; at post-intervention 34 of 36 (94.4%) reached the HCS ceiling of 6, indicating a marked ceiling effect that limits endpoint discrimination and may inflate the apparent magnitude of HCS improvement.

The Friedman omnibus test was significant for every outcome, with Kendall’s W from 0.813 (HCS Total) to 0.950 (SATCo Total). The repeated-measures analysis of variance, reported only as a secondary robustness check, corroborated these results, for the outcomes to which it was applied, with large partial eta-squared values (Table 3).

All pairwise contrasts were significant after Bonferroni adjustment (Table 4). Pre-to-post within-subject effect sizes were large to very large (Cohen’s dz from 1.73 for SATCo Static to 12.67 for HCS Total; the HCS value is inflated by the very small variance of its change scores after data cleaning and should be read together with the bounded Wilcoxon r). Ninety-five percent confidence intervals are reported for all effect sizes (Kendall’s W and Cohen’s d_z_ in Table 3 and Table 4). As a distribution-free estimate of the magnitude of change, the Hodges–Lehmann median difference for the pre-to-post (week 0 to week 8) contrast was 3.0 points (95% CI 3.0 to 3.0) for the HCS, 3.5 (95% CI 3.0 to 4.5) for SATCo Static, 2.0 (95% CI 1.5 to 2.0) for SATCo Active, and 5.5 (95% CI 5.0 to 6.5) for SATCo Total.

## 4. Discussion

This single-arm quasi-experimental study examined within-subject changes following an eight-week structured core muscle strengthening program on head control and segmental trunk control in 36 children with bilateral spastic cerebral palsy at GMFCS levels IV and V. The intervention was associated with statistically significant and consistent improvements across every measured outcome and assessment interval, with large to very large within-subject effect sizes.

### 4.1. Interpretation in Light of Prior Literature

The magnitude of within-group improvement sits at the upper end of the trunk-targeted intervention literature for children with cerebral palsy [20,21,31]. The present study contributes data on the under-represented severely affected non-ambulant phenotype, and the directional consistency of effects across conceptually distinct outcomes is consistent with the proximal-to-distal mechanism postulated in the segmental trunk-control literature [16,19], although formal mediation was not tested.

Several alternative or contributing mechanisms are likely to co-occur rather than to be mutually exclusive, and the single-arm design cannot isolate the intervention effect from them. Concurrent neurodevelopmental maturation over the eight-week window cannot be separated from intervention effects; non-specific therapist engagement and structured thrice-weekly attention may have produced motivational and arousal effects; regression to the mean is possible given selection on incomplete baseline head control; and familiarity with the assessment protocol may have improved cooperation and tolerance for testing. The improvements observed are therefore consistent with a possible intervention effect, but maturation and non-specific factors cannot be ruled out.

### 4.2. Mechanistic Considerations and Head Control Scale Ceiling

The theoretical rationale for the intervention rests on the cephalocaudal hierarchy of postural development. Within this framework, segmental trunk control provides proximal stability that permits, and indeed shapes, the emergence of head stability, anticipatory postural adjustments, and ultimately distal limb function. The present data are partially congruent with this hypothesis: targeted activation of abdominal, paraspinal, and pelvic stabilizers through bridging, quadruped weight-bearing, supported sitting with multidirectional perturbations, and graded pull-to-sit work produced substantial gains in segmental trunk control as measured by the SATCo, and gains were also observed on the HCS. The directional consistency of effects across three conceptually distinct outcome domains lends support to the mediational logic, even though formal mediation analysis was not pre-specified and would require a larger sample with continuous outcome measures.

Several alternative or contributing mechanisms warrant explicit consideration. The first is concurrent neurodevelopmental maturation: children aged 4–10 years continue to undergo neuromuscular development even in the presence of cerebral palsy, and the eight-week study window inevitably captures some maturational change that the single-arm design cannot separate from intervention effects. The second is non-specific therapist engagement and the structured, manualized attention provided to each child three times weekly, which may have produced motivational and arousal effects independent of the specific core-strengthening content. The third is regression to the mean: children were selected on the basis of incomplete head control at baseline, which may have set a lower bound for the baseline measurement that natural variability of test performance would partially correct on retest. The fourth is differential cooperation: as children became familiar with the assessment protocol, their cooperation and tolerance for testing positions may have improved, inflating apparent score gains beyond true postural change. A randomized controlled comparison with an active matched control arm and assessor blinding would be required to disentangle these contributions.

The HCS finding merits separate comment. The 0–6 HCS is comparatively coarse, and with 34 of 36 participants reaching the maximum at week 8, a strong ceiling effect limits discrimination at endpoint and inflates the apparent magnitude of change (notably the very large HCS dz). A finer-grained instrument, such as the Thomas Head Control Scale (0–16), might reveal differentiation that the current scale flattens. Head-control acquisition may also be a comparatively robust capacity in this population given sufficient practice.

### 4.3. Why the Head Control Scale Showed Convergent Endpoints

The Head Control Scale finding merits separate discussion because it is the most clinically counterintuitive result. Two factors plausibly underlie the convergent-endpoint pattern. First, the HCS scoring range used in this study is comparatively narrow (0–6 across three positions × 0–2 each), with ceiling effects becoming relevant once participants approach the maximum. Both subgroups had room at baseline to improve, but the available range constrained the differentiation observable between them at endpoint. A scoring system with finer ordinal granularity—for example, the Thomas Head Control Scale’s 0–4 rating per position, yielding a 0–16 total 24—might have revealed sustained differentiation that the current 0–6 scale flattened. Second, head control acquisition may genuinely be a more developmentally robust capacity in the cerebral palsy population than lower-segmental trunk control, retained even in severely affected children given sufficient practice and time. The slower but ultimately convergent trajectory in GMFCS V participants supports this latter interpretation. Distinguishing these two factors—measurement-instrument ceiling versus underlying biological convergence—will require studies using finer-grained outcome measurement in larger samples across the GMFCS spectrum.

### 4.4. Clinical Implications

Several observations follow from these findings, all of which should be regarded as preliminary and hypothesis-generating. First, structured core muscle strengthening with the parameters used here (eight weeks, three sessions weekly, 45–50 min per session, four-activity manualized rotation) was feasible and well tolerated, with high adherence and no serious adverse events, in this severely affected non-ambulant population. Second, the descriptive differences between GMFCS levels IV and V—whereby children at level V tended to show smaller and later trunk-control gains—are exploratory only; the study was not powered for subgroup comparison, no formal moderation analysis was performed, and these patterns must not be used for prognostic counselling or dose prescription. Third, the SATCo showed floor effects at the lowest segmental levels, so future trials in severe phenotypes should consider complementary outcomes able to capture finer-grained change. Taken together, the clinical implication of this study is limited to establishing feasibility and to justifying an adequately powered, randomized, controlled trial with formal moderation analysis before any inference about clinical implementation, severity-stratified dosing, or individual prognosis can be drawn.

### 4.5. Integration with the International Classification of Functioning, Disability and Health Framework

The outcome set used in this study captures change predominantly at the body-functions-and-structures level of the International Classification of Functioning, Disability and Health framework. The HCS and SATCo characterize specific postural capacities; none directly captures activity-level or participation-level outcomes [32]. While this concentration is appropriate for a study testing a mechanistic-level intervention with a proximal-to-distal hypothesis, it is a deliberate limitation of inference: the data presented here demonstrate that the intervention changes body-function variables in the predicted direction, but do not directly demonstrate that these changes translate into activity-level or participation-level benefits. Whether the observed gains in segmental trunk control translate into improvements in supported sitting tolerance, ease of caregiver handling, communication, feeding safety, or quality of life remains an empirical question for future trials that include activity-level measures and participation-level measures.

### 4.6. Strengths

This study has several methodological strengths. The complete-case dataset with no loss to follow-up over the eight-week intervention period is exceptional in a severely affected pediatric population and eliminates attrition bias from the inferential conclusions. The manualized intervention protocol, with written progression criteria signed off weekly per participant, supports reproducibility and reduces ambiguity about what was actually delivered. The use of standardized outcome measures at all three timepoints by trained physiotherapists supports measurement credibility. The complementary statistical approach—Friedman tests with Wilcoxon post hoc comparisons, parametric repeated-measures ANOVA, and effect-size reporting via both Cohen’s dz and the Wilcoxon r—provides convergent inferential evidence and addresses concerns about the ordinal nature of the outcomes.

### 4.7. Limitations

Several limitations qualify the interpretation of these findings. The most consequential is the absence of a randomized control arm, which precludes causal attribution of the within-group improvements to the intervention rather than to maturation, regression to the mean, non-specific therapist effects, or assessment learning; the very large effect sizes reduce but do not eliminate this concern. Assessors were not blinded and scored live, and no in-study reliability check was performed. The ordinal outcomes and their ceiling/floor properties limit sensitivity. Participants were recruited at two centers in one city from families able to commit to thrice-weekly attendance for eight weeks, a motivated and likely higher-resource sample, so generalizability to lower-resource or rural settings is uncertain. No post-intervention follow-up was conducted, leaving maintenance of gains unknown. Removal of two out-of-criterion participants at the data-cleaning stage reduced the sample to 36; sensitivity of the findings to this decision was limited because the direction and significance of all results were unchanged. Fidelity was scored dichotomously at the item level rather than on a graded scale, which limits sensitivity to minor deviations in delivery. Finally, no minimal clinically important difference has been established for the HCS or the SATCo in this population; the statistical changes reported here should therefore be interpreted as measured improvements of currently undetermined clinical importance, pending anchor-based responsiveness studies.

### 4.8. Future Research Directions

Five priorities follow from these findings. First, an adequately powered randomized controlled trial comparing the present intervention to a dose-matched conventional physiotherapy comparator is warranted; the within-group effect sizes observed here would yield realistic between-group effect-size estimates in the range of d ≈ 0.4 to 0.7 after accounting for the active-control gains expected from conventional physiotherapy, requiring approximately 35 to 65 participants per arm. Second, recruitment of a broader severity range—extending to include GMFCS levels II and III alongside IV and V—would allow formal severity-stratified moderator analysis across the full ambulant-to-non-ambulant spectrum. Third, the inclusion of instrumented mechanistic outcomes (surface electromyography of paraspinal and abdominal musculature, three-dimensional kinematics of head and trunk angular response, pressure-platform measures of supported sitting) would test the proximal-to-distal hypothesis directly and provide continuous outcomes less subject to ceiling and floor effects. Fourth, longer-term follow-up at three and six months post-intervention would establish whether biomechanical gains are maintained, decay, or progress, addressing the maturation question that single-arm or short-follow-up designs cannot resolve. Fifth, integration of activity-level (Gross Motor Function Measure–66, GMFM-66) and participation-level (Caregiver Priorities and Child Health Index of Life with Disabilities, CPCHILD) outcomes would address the International Classification of Functioning translation gap noted above and would establish whether the substantial body-function gains observed in this study translate into family-perceived improvements in daily life.

## 5. Conclusions

An eight-week, manualized core muscle strengthening program delivered three times weekly was associated with statistically significant and consistent within-subject improvements in head control and segmental trunk control in 36 children with bilateral spastic cerebral palsy at GMFCS levels IV and V, with large to very large within-subject effect sizes. Because the study lacked a control group and post-intervention follow-up, these findings should not be interpreted as supporting routine clinical adoption; they support further controlled investigation of structured trunk-targeted training in this population, with an adequately powered randomized controlled trial as the next definitive step.

## Figures and Tables

**Table 1:** Baseline demographic and clinical characteristics of the analyzed cohort (N = 36), overall and by GMFCS level.

Characteristic	GMFCS IV (n = 19)	GMFCS V (n = 17)	All (N = 36)
Age, years, mean ± SD	6.9 ± 2.2	7.0 ± 1.9	6.94 ± 2.01
Sex, male/female, n	11/8	9/8	20/16
Distribution, quad/di, n	10/9	17/0	27/9
Baseline HCS, mean ± SD	3.00 ± 0.00	3.00 ± 0.00	3.00 ± 0.00
Baseline SATCo Total, mean ± SD	1.37 ± 0.68	0.94 ± 0.24	1.17 ± 0.56

quad = quadriplegia; di = diplegia; HCS = Head Control Scale; SATCo = Segmental Assessment of Trunk Control. Additional clinical descriptors (epilepsy, feeding status, communication and cognition, orthotic use, seating system, and scoliosis severity) were not collected as discrete study variables because every participant was medically stable throughout the study period, with no change in these conditions over the eight weeks; children with uncontrolled seizures, recent botulinum toxin, or fixed scoliosis exceeding 40 degrees were excluded a priori.

**Table 2:** Descriptive statistics for head and trunk control outcomes at each assessment timepoint (N = 36).

Outcome	Timepoint	Mean ± SD	Median [IQR]	Range
HCS	Week 0	3.00 ± 0.00	3.0 [3.0, 3.0]	3–3
	Week 4	4.56 ± 1.34	5.0 [3.0, 6.0]	3–6
	Week 8	5.94 ± 0.23	6.0 [6.0, 6.0]	5–6
SATCo Static	Week 0	1.17 ± 0.56	1.0 [1.0, 1.0]	0–3
	Week 4	2.53 ± 1.36	2.0 [1.8, 3.0]	1–7
	Week 8	4.83 ± 2.29	6.0 [2.0, 7.0]	2–7
SATCo Active	Week 0	0.00 ± 0.00	0.0 [0.0, 0.0]	0–0
	Week 4	0.67 ± 0.48	1.0 [0.0, 1.0]	0–1
	Week 8	1.89 ± 0.78	2.0 [1.0, 2.2]	1–3
SATCo Total	Week 0	1.17 ± 0.56	1.0 [1.0, 1.0]	0–3
	Week 4	3.19 ± 1.62	3.0 [1.8, 4.0]	1–7
	Week 8	6.72 ± 2.98	8.0 [3.0, 9.2]	3–10

SD = standard deviation; IQR = interquartile range; HCS = Head Control Scale; SATCo = Segmental Assessment of Trunk Control.

**Table 3:** Omnibus tests for change over time across the three assessment timepoints (N = 36).

Outcome	Friedman χ^2^ (df = 2)	*p*-Value	Kendall’s W (95% CI)	RM-ANOVA F (df = 2, 70)	*p*-Value	Partial η^2^
HCS Total	58.51	<0.001	0.813 (95% CI 0.784–0.856)	133.00	<0.001	0.792
SATCo Static	67.05	<0.001	0.931 (95% CI 0.899–0.961)	90.83	<0.001	0.722
SATCo Active	65.44	<0.001	0.909 (95% CI 0.876–0.943)	NA	NA	NA
SATCo Total	68.40	<0.001	0.950 (95% CI 0.934–0.975)	125.84	<0.001	0.782

Partial η^2^ benchmarks: small ≈ 0.01, medium ≈ 0.06, large ≥ 0.14. Sphericity was assumed; Mauchly’s test was non-significant for the outcomes analyzed parametrically. NA = not applicable (parametric modelling was not performed for the SATCo Active subscale, owing to its zero-variance baseline floor). RM-ANOVA = repeated-measures analysis of variance.

**Table 4:** Within-subject pairwise comparisons between assessment timepoints (Wilcoxon signed-rank tests; Bonferroni-adjusted α = 0.017; N = 36).

Outcome	Comparison	Mean Change ± SD	Z	*p*-Value	Cohen’s dz (95% CI)	Wilcoxon r
HCS	Week 0 → Week 4	1.56 ± 1.34	4.23	<0.001	1.16 [0.85, 1.64]	0.71
	Week 0 → Week 8	2.94 ± 0.23	5.86	<0.001	12.67 [8.16, 17.83]	0.98
	Week 4 → Week 8	1.39 ± 1.29	4.29	<0.001	1.07 [0.82, 1.44]	0.72
SATCo Static	Week 0 → Week 4	1.36 ± 1.22	4.63	<0.001	1.11 [0.88, 1.52]	0.77
	Week 0 → Week 8	3.67 ± 2.12	5.28	<0.001	1.73 [1.42, 2.25]	0.88
	Week 4 → Week 8	2.31 ± 1.47	5.15	<0.001	1.57 [1.30, 1.99]	0.86
SATCo Active	Week 0 → Week 4	0.67 ± 0.48	4.90	<0.001	1.39 [0.99, 2.01]	0.82
	Week 0 → Week 8	1.89 ± 0.78	5.31	<0.001	2.41 [2.10, 2.94]	0.88
	Week 4 → Week 8	1.22 ± 0.64	5.24	<0.001	1.92 [1.50, 2.61]	0.87
SATCo Total	Week 0 → Week 4	2.03 ± 1.50	4.61	<0.001	1.35 [1.03, 1.88]	0.77
	Week 0 → Week 8	5.56 ± 2.81	5.25	<0.001	1.98 [1.66, 2.52]	0.88
	Week 4 → Week 8	3.53 ± 1.84	5.26	<0.001	1.91 [1.64, 2.36]	0.88

Z = standardized Wilcoxon signed-rank statistic (normal approximation); *p* = two-tailed (*p* < 0.001). The signed-rank T equalled zero for every contrast because all non-zero within-subject differences were positive; this reflects uniformly directional improvement and does not imply identical change scores. SD = standard deviation; dz = Cohen’s d for paired samples; r = Wilcoxon effect size (|Z|/√N). 95% confidence intervals for d_z_ were obtained by bias-corrected bootstrap (5000 resamples); those for Kendall’s W (Table 3) by the same method.

## Data Availability

The data presented in this study are available on request from the corresponding author due to privacy.

## Supplementary Materials

The following supporting information can be downloaded at: https://www.mdpi.com/article/10.3390/healthcare14142170/s1.

## Abbreviations

The following abbreviations are used in this manuscript:

CP	Cerebral Palsy
CPCHILD	Caregiver Priorities and Child Health Index of Life with Disabilities
DSR	Deanship of Scientific Research
GMFCS	Gross Motor Function Classification System
GMFM-66	Gross Motor Function Measure–66
HCS	Head Control Scale
ICF	International Classification of Functioning, Disability and Health
SATCo	Segmental Assessment of Trunk Control
SPSS	Statistical Package for the Social Sciences
TIDieR	Template for Intervention Description and Replication
TREND	Transparent Reporting of Evaluations with Nonrandomized Designs
WAQF	KAU Endowment
