# Supplementary material for: Preliminary Changes in Head Control and Segmental Trunk Control Following an Eight-Week Core Strengthening Program in Children with Bilateral Spastic Cerebral Palsy
Source: Healthcare (Basel). 2026 Jul 18;14(14):2170. doi: 10.3390/healthcare14142170 (PMC13411832; doi:10.3390/healthcare14142170)
Supplement: Supplementary file 1 [file healthcare-14-02170-s001.zip › healthcare-4398078-supplementary.pdf]

Supplementary File S1: Sensitivity Analysis Including All Enrolled Participants (N = 38)

This sensitivity analysis repeats the primary analyses on the full enrolled sample of 38 children—that is, including the two participants who were removed from the main analysis at the data-cleaning stage because they had full head control on the supported-sitting item at baseline (contrary to the eligibility criterion). As shown below, the direction and statistical significance of every result are unchanged relative to the primary analysis (N = 36) reported in the main text. As in the main analysis, parametric repeated-measures ANOVA was not applied to the SATCo Active subscale, whose baseline floor (mean 0.00, SD 0.00) precludes meaningful parametric modelling.

Table S1. Descriptive statistics for head and trunk control outcomes at each assessment timepoint (N = 38).

Outcome	Timepoint	Mean ± SD	Median [IQR]	Range
HCS	Week 0	3.11 ± 0.51	3.0 [3.0, 3.0]	3–6
	Week 4	4.58 ± 1.33	5.0 [3.0, 6.0]	3–6
	Week 8	5.92 ± 0.27	6.0 [6.0, 6.0]	5–6
SATCo Static	Week 0	1.21 ± 0.74	1.0 [1.0, 1.0]	0–4
	Week 4	2.55 ± 1.50	2.0 [1.2, 3.0]	0–7
	Week 8	4.79 ± 2.34	6.0 [2.0, 7.0]	1–7
SATCo Active	Week 0	0.00 ± 0.00	0.0 [0.0, 0.0]	0–0
	Week 4	0.63 ± 0.49	1.0 [0.0, 1.0]	0–1
	Week 8	1.92 ± 0.85	2.0 [1.0, 2.8]	1–4
SATCo Total	Week 0	1.21 ± 0.74	1.0 [1.0, 1.0]	0–4
	Week 4	3.18 ± 1.72	3.0 [1.2, 4.0]	0–7
	Week 8	6.71 ± 3.08	8.0 [3.0, 9.8]	2–11

SD = standard deviation; IQR = interquartile range; HCS = Head Control Scale; SATCo = Segmental Assessment of Trunk Control.

Table S2. Omnibus tests for change over time (N = 38).

Outcome	Friedman χ^2 (df = 2)	p	Kendall's W	RM-ANOVA (2, 74)	p	ηp^2
HCS Total	60.05	< .001	0.790	118.06	< .001	0.761
SATCo Static	70.69	< .001	0.930	92.33	< .001	0.714
SATCo Active	68.84	< .001	0.906	n/a	n/a	n/a
SATCo Total	72.06	< .001	0.948	131.52	< .001	0.780

n/a = not applicable (parametric modelling was not performed for the SATCo Active subscale, owing to its zero-variance baseline floor). Non-parametric Friedman tests and Kendall's W cover all four outcomes. RM-ANOVA = repeated-measures analysis of variance.

Table S3. Within-subject pairwise comparisons between timepoints (Wilcoxon signed-rank tests; Bonferroni-adjusted α = .017; N = 38).

Outcome	Comparison	Mean change ± SD	Z	p	Cohen's dz	r
HCS	Wk 0 → Wk 4	1.47 ± 1.35	4.23	< .001	1.09	0.69
	Wk 0 → Wk 8	2.82 ± 0.61	5.88	< .001	4.63	0.95
	Wk 4 → Wk 8	1.34 ± 1.28	4.38	< .001	1.05	0.71
SATCo Static	Wk 0 → Wk 4	1.34 ± 1.21	4.71	< .001	1.11	0.76
	Wk 0 → Wk 8	3.58 ± 2.11	5.41	< .001	1.69	0.88
	Wk 4 → Wk 8	2.24 ± 1.46	5.31	< .001	1.53	0.86
SATCo Active	Wk 0 → Wk 4	0.63 ± 0.49	4.90	< .001	1.29	0.79
	Wk 0 → Wk 8	1.92 ± 0.85	5.45	< .001	2.26	0.88
	Wk 4 → Wk 8	1.29 ± 0.77	5.38	< .001	1.68	0.87
SATCo Total	Wk 0 → Wk 4	1.97 ± 1.50	4.69	< .001	1.32	0.76
	Wk 0 → Wk 8	5.50 ± 2.81	5.39	< .001	1.96	0.88
	Wk 4 → Wk 8	3.53 ± 1.83	5.41	< .001	1.93	0.88

Z = standardized Wilcoxon signed-rank statistic (normal approximation); p = two-tailed (all exact p < .001); dz = Cohen's d for paired samples; r = Wilcoxon effect size (|Z|/√N).

Conclusions. Including the two data-cleaning cases (N = 38) leaves every conclusion unchanged: all omnibus tests remain significant (all p < .001), all pairwise contrasts remain significant after Bonferroni adjustment, and the effect-size estimates are materially the same as in the primary N = 36 analysis. The removal of the two participants therefore did not influence the study's findings.
